# Proteinuria—take a closer look!

**DOI:** 10.1007/s00467-019-04454-w

**Published:** 2020-01-10

**Authors:** Arend Bökenkamp

**Affiliations:** Department of Pediatric Nephrology, Emma Children’s Hospital, Amsterdam University Medical Center, Vrije Universiteit, De Boelelaan 1112, NL-1081 HV Amsterdam, The Netherlands

**Keywords:** Proteinuria, Low molecular weight proteins, Tubulointerstitial disease, Glomerular disease, Acute kidney injury, Selectivity

## Abstract

Proteinuria is a hallmark of kidney disease. Therefore, measurement of urine protein content plays a central role in any diagnostic work-up for kidney disease. In many cases, proteinuria analysis is restricted to the measurement of total protein content knowing that very high levels of proteinuria (nephrotic proteinuria) are characteristic of glomerular disease. Still, proteinuria can also be a manifestation of impaired tubular protein reabsorption or even be physiological. This review will discuss the physiology of renal protein handling and give guidance on a more sophisticated analysis of proteinuria differentiating albumin, low-molecular weight proteins and immunoglobulins. These non-invasive tests are available in most routine clinical laboratories and may guide the clinician in the diagnostic process before ordering far more expensive (molecular genetic testing) and/or invasive (kidney biopsy) diagnostics.

## Introduction

Besides serum creatinine, blood pressure, and urinalysis, the measurement of urinary protein excretion plays a central role in the recognition and classification of renal disease. Even small amounts of proteinuria, i.e., microalbuminuria, are associated with dismal outcomes and are therefore included in the staging of chronic kidney disease according to the KIDGO guidelines [1]. This is even more so for nephrotic range proteinuria. As the intact glomerular filter is almost impermeable to large proteins, proteinuria is a hallmark of glomerular disease. Still, significant proteinuria can also be found in tubulointerstitial disease, which can pose a diagnostic challenge. This is illustrated in the case presented by Preston et al. [2] in this issue of *Pediatric Nephrology*. The paper by Beara-Lasic et al. [3] also published in this issue demonstrates that a more detailed analysis of urinary protein excretion can distinguish glomerular from tubulointerstitial disease and pure tubular proteinuria. Of note, their approach only requires measurement of α1-microglobulin on top of the standard parameters, i.e., urinary albumin, total protein, and creatinine. The present review will put their findings in a broader perspective and focus on the physiology and diagnostic potential of low-molecular weight (LMW) proteins in the urine. It will not address albuminuria in detail, a finding which has received much more attention and been extensively reviewed elsewhere [4–11].

## Filtration and reabsorption of plasma proteins

Under normal circumstances, urine is almost free of protein (i.e., proteinuria < 4 mg/m^2^/h or protein-creatinine ratio of < 180 mg/g (20 mg/mmol)). Still, there are three situations when proteinuria may be physiological: (i) orthostatic proteinuria [12], (ii) febrile proteinuria, and (iii) exercise proteinuria [13, 14]. In all these situations, proteinuria is transient and hence must be absent when tested in a first morning urine sample collected directly after getting up, after recovery from the febrile condition, or after recovery from strenuous exercise, respectively.

**Fig. 1:**
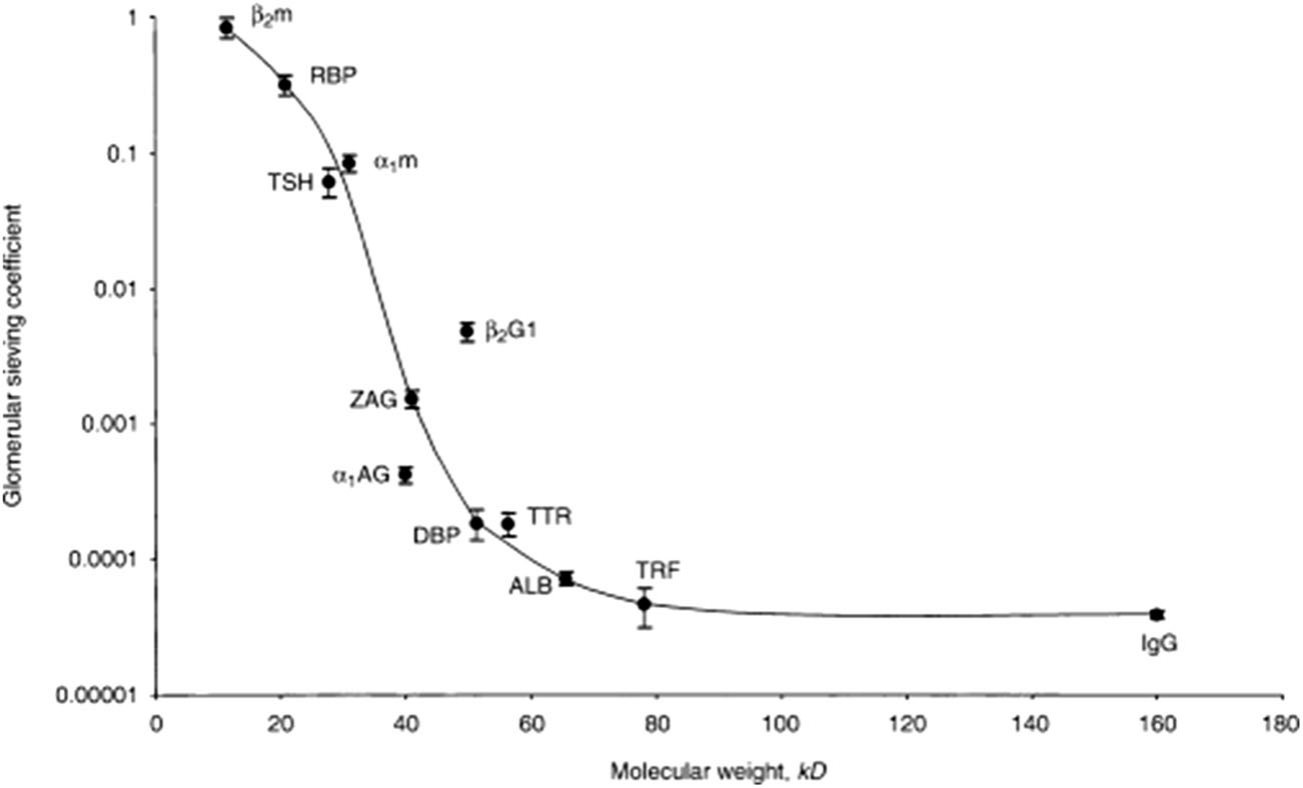
Estimated glomerular sieving coefficients for 12 plasma proteins versus molecular weight. Abbreviations: ß2m, ß2-microglobulin; RBP, retinol-binding protein; α1m, α1-microglobulin; TSH, thyroid-stimulating hormone; ß2GI, ß2-glycoprotein-I; ZAG, zinc-α2-globulin; α1AG, α1-acid glycoprotein; DBP, vitamin D-binding protein; TTR, transthyretin; ALB, albumin; TRF, transferrin; IgG, immunoglobulin G. From Norden et al. [15], reproduced with permission

**Fig. 2:**
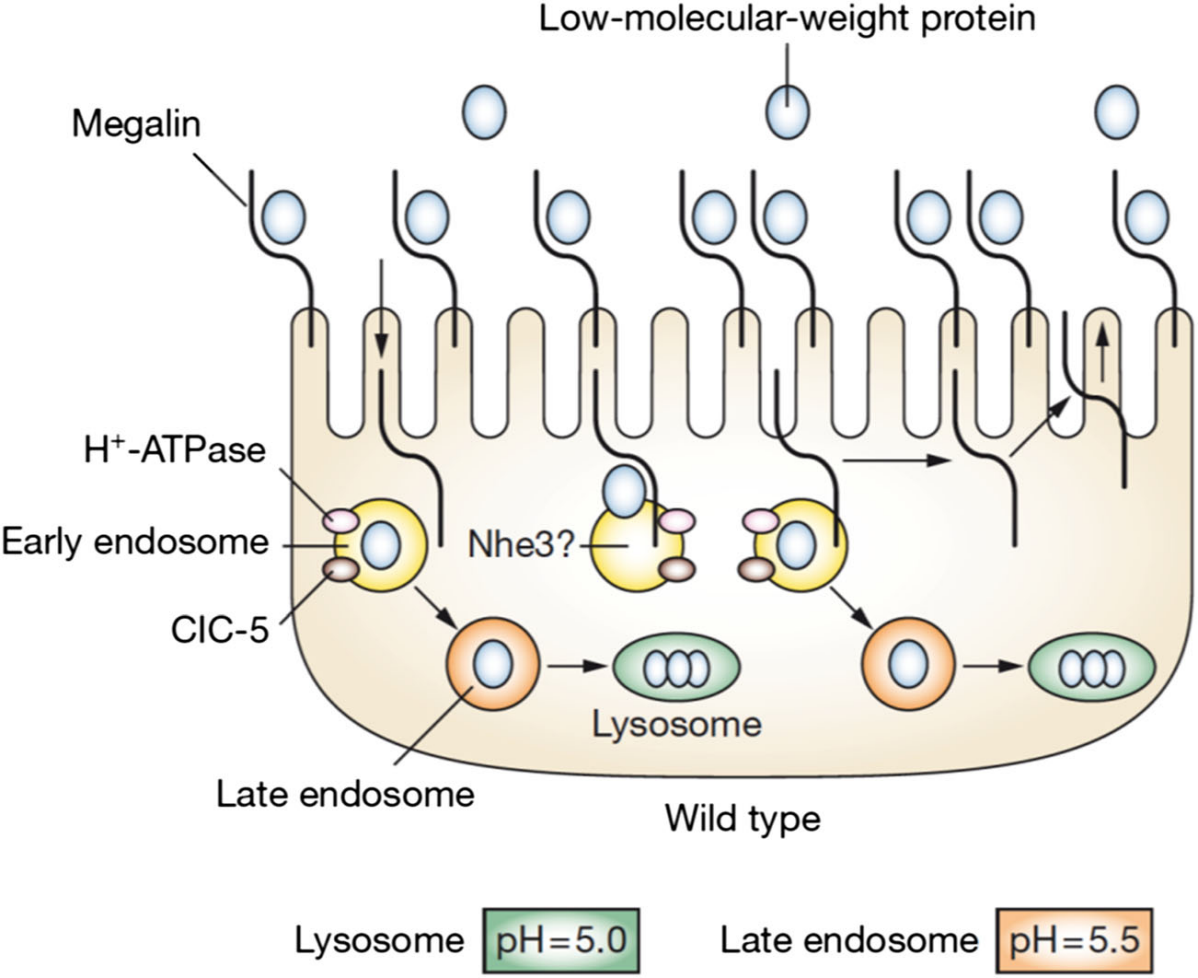
Absorption and intra-cellular handling of LMW proteins in the proximal tubule. Megalin is present on the cell surface in high abundance. When low-molecular-weight proteins bind to megalin, this “cargo” is internalized into the early endosome. The endosome is acidified by the action of H^+^ATPase in concert with chloride channel 5 (CLCN5). At low pH, the cargo dissociates from megalin and passes through the late endosome and on to the lysosome where it is degraded. Megalin returns from the early endosome back to the surface of the cell, where it is available for internalization of more cargo. From Guggino [16], reproduced with permission

Water and small solutes up to the size of inulin (5 kDa) can pass the glomerular filter freely. For larger molecules, permeability is inversely related to molecular size. Therefore, LMW proteins with a molecular mass between 10 and 20 kDa such as α1-microglobulin, β-2 microglobulin, cystatin C, retinol-binding protein (RBP), and many other macromolecules including hormones and cytokines also pass the glomerular filter in considerable amounts (Fig. 1). Still, the final urine contains negligible amounts of LMW proteins. This is due to the extensive reabsorption of proteins in the proximal tubule by receptor-mediated multi-ligand endocytosis involving megalin and cubulin. Reabsorbed LMW proteins are digested at low pH in lysosomes in the proximal tubule and do not enter the circulation intact (Fig. 2) [16].

**Fig. 3:**
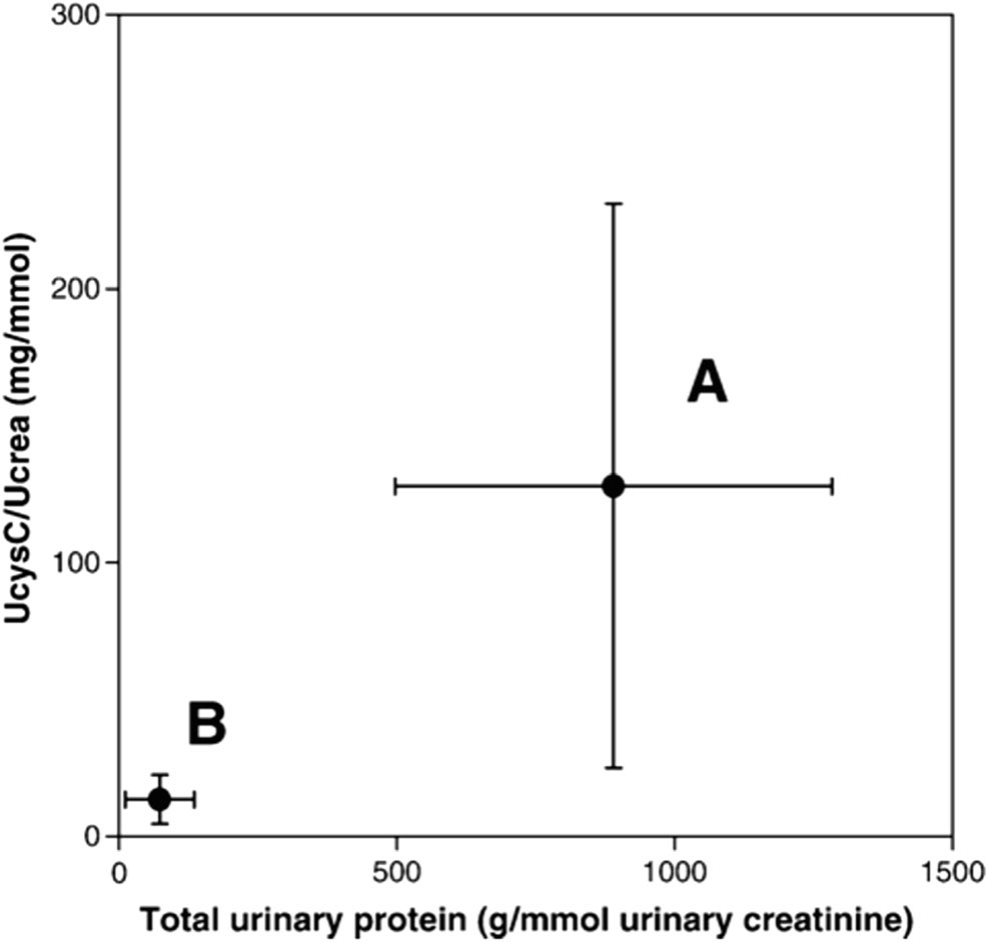
Simultaneous changes of the urinary cystatin C-creatinine ratio and the protein-creatinine ratio. Paired measurement of patients during the active phase (A) and in remission of minimal change nephropathy (B). Data presented as mean ± SD. From Herget-Rosenthal et al. [18], reproduced with permission

Although the plasma concentrations of LMW proteins are in the mg/l range, i.e., almost 1000 times lower than albumin, the higher permeability leads to some 9.6 g being filtered and reabsorbed each day in an adult [17]. This process is saturable if excessive amounts of proteins are filtered, leading to shedding of LMW proteins in the absence of tubular damage. This is exemplified when comparing urinary cystatin C excretion in minimal change nephrotic syndrome during recurrence and in remission (Fig. 3) [18]. This overflow LMW proteinuria has also been reported for other LMW proteins [19, 20].

**Fig. 4:**
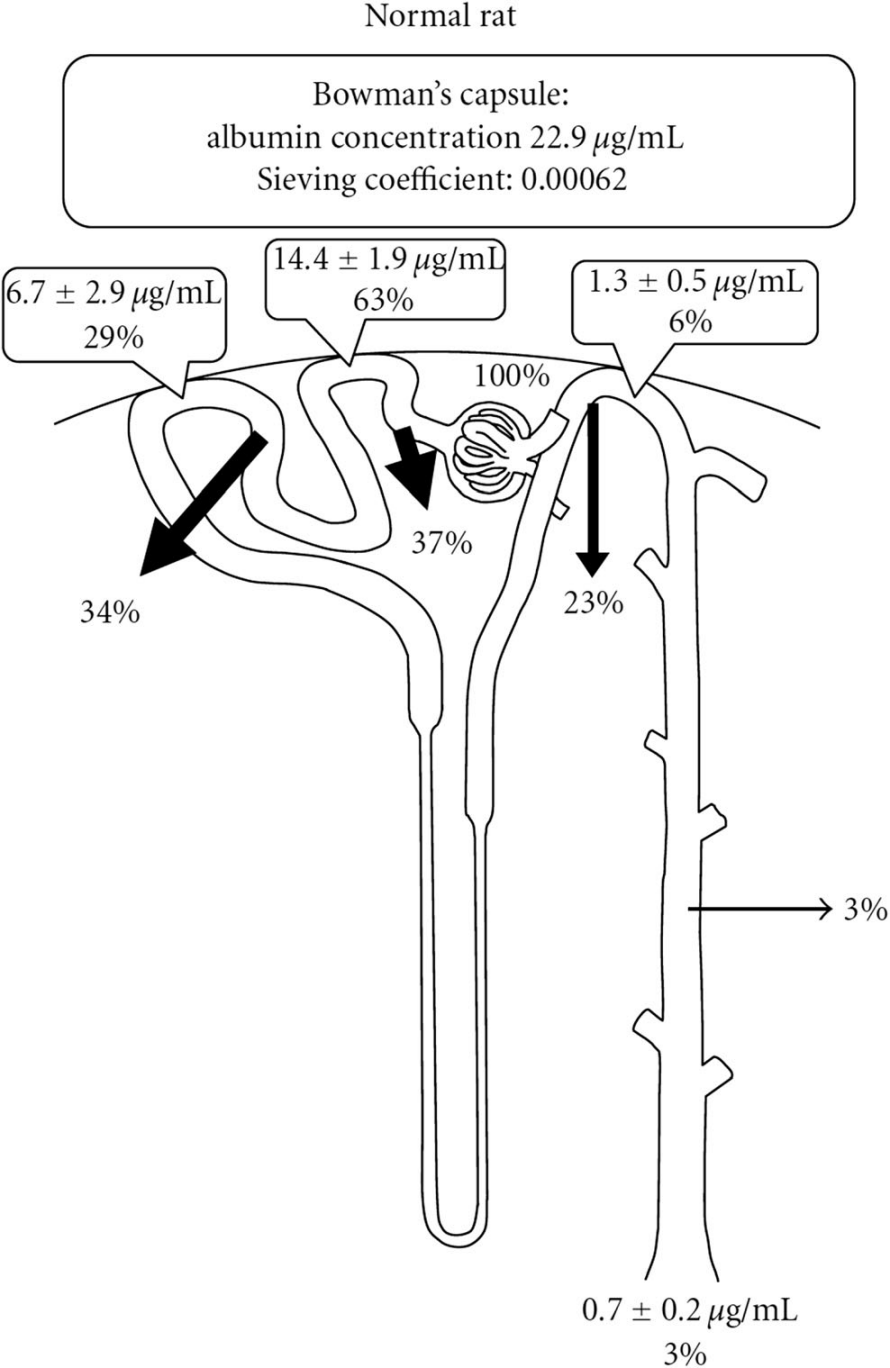
Albumin concentration along the nephron. Data calculated from a rat fractional micropuncture study. Arrows indicate the percentage of ultrafiltrated albumin which is reabsorbed in the respective nephron segment. From Tojo et al. [17], reproduced with permission

By contrast, the intact glomerular membrane is almost impermeable to albumin due its larger size and negative charge causing reflection of this anionic molecule [21–23]. In rat models, the sieving coefficient of albumin (i.e., the albumin-concentration in ultrafiltrate/plasma albumin-concentration) has been determined at about 0.0001 to 0.0006 [17]. This results in around 3.3 g of albumin being filtered per day in an adult [17]. Based on micropuncture studies in rats [24], 71% is reabsorbed in the proximal and 26% in the distal tubule so that albumin excretion is negligible under normal conditions (Fig. 4). More recently, much higher glomerular sieving coefficients around 0.02 have been reported for albumin and molecules of similar size. Dickson et al. propose that filtered albumin is not only reabsorbed in clathrin-coated pits on the surface of proximal tubular cells following binding to cubulin but also via fluid-phase endocytosis [25]. They hypothesize that absorbed albumin can leave the cell *intact* after binding to the neonatal Fc receptor (FcRn) rather than being degraded in lysosomes.

Both the classical view and Dickson’s findings imply that substantial amounts of albumin will be detected in the urine of individuals with defective tubular protein reabsorption but normal glomeruli, and does not necessarily imply a glomerular origin of albuminuria. This is illustrated by an increased urine albumin-creatinine ratio of 38 mg/mmol in patients with Dent disease, who have impaired proximal tubular protein absorption [16, 26], reported by Norden et al. [27].

## Proteinuria

Pathological proteinuria may result from two principal mechanisms (or a combination of the two): (i) excessive permeability of the glomerular barrier for protein or (ii) impaired reabsorption of protein in the proximal tubule. While there is an association between nephrotic range proteinuria and glomerular disease, there is considerable overlap with non-glomerular disease which can also cause large proteinuria and albuminuria [28].

## Measurement of proteins in the urine

The first screening for proteinuria is by urine dipstick. This colorimetric method is based on a change in pH in the presence of *anionic* proteins, i.e., albumin and transferrin, while most other proteins have much less affinity for protons. Therefore, the limits of detection vary considerably between different proteins: 150 mg/l for albumin, 200 mg/l for transferrin, 500 mg/l for IgG, 600 mg/l for ß2-microglobulin, and > 1000 mg/l for immunoglobulin light chains [29]. Urine dipsticks have good sensitivity as screening tool for macroalbuminuria (albumin-creatinine ratio > 30 mg/mmol), yet specificity is limited [30]. It should be borne in mind that the dipstick measures urine protein *concentration* and (anti-) diuresis therefore strongly influences sensitivity and specificity of this test.

In most clinical laboratories, total protein is measured using the colorimetric biuret [31] or a turbidimetric method [32] and related to urine creatinine to correct for urine concentration when using spot urine samples. Urine proteins can be differentiated using SDS-PAGE gel-electrophoresis and fast protein liquid chromatography discriminating between glomerular and tubular proteinuria [33]. Still, in daily routine, a selection of marker proteins is used to classify proteinuria [34, 35]. More recently, a mass spectrometry–based proteonomic analysis of urine was introduced in research settings [36].

In order to achieve high sensitivity (“microalbuminuria”), urine albumin concentrations are measured by immunoturbidimetry or nephelometry using anti-albumin antibodies [37]. This method is also used for the measurement of LMW proteins such as α1-microglobulin, ß2-microglobulin, retinol-binding protein (RBP), and cystatin C. The characteristics and upper limits of normal of the different marker proteins are summarized in Table 1. It should be borne in mind that these reference values do not apply to neonates, where higher values apply due to tubular immaturity and lower creatinine excretion [42–44]. α1-microglobulin and RBP are preferred above ß2-microglobulin because of the instability of the latter in acidic urine [45]. Branten et al. described a method for alkalizing urine by oral bicarbonate administration before urine collection to make sure that urine pH is above six [46], yet it is doubtful if this method is suitable for daily clinical practice, in particular in children. Data from Tomlinson suggest that RBP is most closely associated with histologically proven tubular abnormality and least affected by increasing albuminuria [20].

**Table 1:** Characteristics of different LMW proteins in urine. Upper limits for adults are 90th centiles [38], limits for children refer to the maximum from 43 healthy controls [39]. For conversion to SI units (mg/mmol) divide by 9

LMW protein	Molecular mass [40]	Upper reference limit	Interactions
α1-microglobulin	26 kDa	11.7 mg/g creatinine, adults [38] 19.8 mg/g creatinine, children [39]	None
ß2-microglobulin	12 kDa	2.9 mg/g creatinine, adults [38] 0.37 mg/g creatinine, children [39]	Unstable at pH < 6
Cystatin C	13 kDa	0.77 mg/g creatinine, not specified [41]	None
Retinol-binding protein	22 kDa	0.1 mg/g creatinine, adults [38] 0.22 mg/g creatinine, children [39]	None

As stated above, urine protein concentration is strongly influenced by (anti-)diuresis. Therefore, proteinuria is quantified either in timed urine samples or by normalizing for urine creatinine concentration as a surrogate marker of (anti-)diuresis [47]. The former is hampered by inaccurate urine collection [47, 48] while the latter assumes normal creatinine production [49]. Conditions with increased (e.g., body building, creatinine supplements) or decreased production (e.g., neuromuscular disease, muscle wasting) will lead to falsely decreased or increased ratios, respectively. This is illustrated by data from Carter et al. who showed that intra-individual variability improved when urine concentrations were normalized for urine creatinine, while inter-individual variability did not improve or even increased [50].

Studies comparing both methods for albuminuria and total proteinuria suggest that analysis of spot urine is sufficiently accurate in clinical practice [47, 51, 52]. However, Lane et al. noted a logarithmic relationship between spot protein-creatinine ratio and 24-h protein excretion and concluded that spot urine analysis is less suitable for the follow-up of high proteinuria [51]. Hogan et al. found a relatively poor correlation between both parameters [53]. Therefore, a recent KDIGO conference on glomerular disease recommended 24-h measurements when changes in proteinuria impact therapeutic decisions [54].

The commonly used unit to express the protein-creatinine ratio is gram/gram, and this can be transformed to SI units (g/mmol) by dividing by 9.

## Assessing selectivity of glomerular proteinuria

In heavy glomerular proteinuria, the selectivity index (SI) describes if urine protein is largely composed of albumin and transferrin (“selective”) or if significant amounts of very large proteins, such as IgG, are present too. The SI is calculated as the relation of IgG in blood and urine related to transferrin (uIgG × sTf / sIgG × uTf) [55]. An SI ≤ 0.10 is classified as selective proteinuria, a typical finding in minimal change disease, and bears a good prognosis. An SI between 0.11 and 0.20 is classified as moderately selective and ≥ 0.21 as unselective, often observed in steroid-resistant nephrotic syndrome with ominous prognosis of kidney function [56]. In patients with moderately selective and unselective proteinuria, an increased fractional excretion of α1-microglobulin indicates a worse prognosis reflecting additional tubulointerstitial damage (see below). McQuarrie et al. measured the fractional excretions of albumin (FE_Alb_) and IgG (FE_IgG_) [57]. In their hands, both FE_Alb_ (hazard ratio 35.2 using a cutoff 0.0325%) and FE_IgG_ (hazard ratio 37.1 using a cutoff 0.043%) were strong predictors of end-stage kidney disease with a median follow-up of 7 years.

## Low-molecular weight proteinuria in glomerular disease

Several authors have reported LMW proteinuria in patients with documented glomerular disease [19, 56, 58–62]. Portman et al. measured the fractional excretion of ß2-microglobulin in children with tubular and glomerular disease [58]. While they observed a highly significant difference between both groups (0.104 vs. 4.27%) they noted that about one half of the patients with glomerular disease also had increased ß2-microglobulinuria. Re-assessment for the presence of tubulointerstitial lesions on renal biopsy showed that increased excretion of ß2-microglobulin separated the 13 patients with such lesions from 17 patients with isolated glomerular findings (3.76 vs. 0.063%). They suggest a cutoff of 0.36% to discriminate between isolated glomerular and glomerular disease with tubulointerstitial damage.

Van den Brand et al. studied LMW-protein markers ß2-microglobulin and α1-microglobulin as predictors of disease progression in idiopathic membranous glomerulopathy [59, 63]. They noted that the prognostic performance of either marker (area under the receiver-operating characteristic curve (AUROC)) was around 0.80 and comparable to the Toronto Risk Score, which incorporates baseline GFR, GFR-slope during 6-month follow-up, and persistent proteinuria [63]. A more detailed analysis of the risk score revealed that baseline GFR and change in GFR, rather than the severity of proteinuria, predicted deterioration of kidney function. Although not documented histologically, their findings suggest that the prognostic value of the LMW protein markers in this setting reflects tubulointerstitial damage rather than impaired reabsorption due to overflow proteinuria. Although urinary ß2-microglobulin was related to kidney function in patients with IgA nephropathy, Shin et al. did not find a correlation between urinary ß2-microglobulin and tubulointerstitial inflammation or fibrosis [62].

Several papers have addressed LMW proteinuria as a potential predictor of steroid resistance in childhood nephrotic syndrome [19, 60, 61]. Sesso et al. measured RBP and ß2-microglobulin at presentation in 37 patients with idiopathic nephrotic syndrome [61]. In their hands, both markers were much more elevated in steroid-resistant patients: a ß2-microglobulin-creatinine ratio > 3 mg/g and a RBP-creatinine ratio > 4 mg/g were 3.0 and 3.8 times more likely to come from steroid-unresponsive patients, respectively. By contrast, Valles et al. observed comparable excretion of urinary ß2-microglobulin in relapse in patients with steroid-dependent and steroid-resistant nephrotic syndrome [19]. In 11 patients with focal segmental glomerulosclerosis (FSGS), they found no significant correlation between urinary ß2-microglobulin and tubulointerstitial damage (*r* = 0.54, *p* = 0.19)

## Low-molecular weight proteinuria as marker of acute kidney injury

In recent years, a number of urine markers have been identified for the prediction of imminent acute renal failure. These include neutrophil gelatinase-associated lipocalin (NGAL) [64] and kidney injury molecule-1 (KIM-1) [65], which are upregulated in damaged tubular cells. Proximal tubular dysfunction in acute kidney injury (AKI) leads to impaired reabsorption of LMW proteins and has therefore been studied as a marker/predictor of AKI. Of all LMW protein markers, cystatin C has been studied most extensively. Herget-Rosenthal et al. addressed non-oliguric AKI in an adult ICU setting [40]. In their hands, increased excretion of cystatin C and α1-microglobulin was a strong predictor (AUROC 0.92 and 0.86, respectively) for the need to initiate renal replacement therapy (RRT) within a median interval of 4 days. Cutoffs with optimal sensitivity and specificity were 9 mg/g for cystatin C and 180 mg/g for α1-microglobulin. Koyner et al. [66] studied adult patients following cardiac surgery and classified AKI using the RIFLE criteria [67]. The urinary cystatin C-creatinine ratio at the end of cardio-pulmonary bypass, on admission to the ICU and at 6 h after admission, was significantly higher in patients who developed AKI and even higher in patients requiring RRT when compared to patients with an uneventful course. In this series, the AUROC to predict AKI was 0.734.

Carter et al. found high inter- and intra-individual variability of serum and urine markers of AKI in patients with chronic kidney disease. However, the changes during AKI were high, indicating that these markers are still clinically useful if baseline values are available [50]. For urine markers, normalization to creatinine concentration reduced intra-individual variability.

A recent meta-analysis of four studies in children showed an AUROC of 0.85 (95% CI 0.81–0.88) for urinary cystatin C [68], while this marker was less accurate for the prediction of AKI in adults (AUROC 0.64, 95% CI 0.62–0.66) [69]. This meta-analysis was hampered by heterogeneity across the studies, in particular, lack of exclusion of pre-renal azotemia in many studies [69]. Assessing RRT as an outcome parameter in their meta-analysis, Klein et al. found a better predictive value for urinary cystatin C (AUROC 0.72, 95% CI 0.575–0.868), which improved to 0.790 (0.645 to 0.934) after normalization for creatinine [70], stressing the need to normalize LMW proteins for urine creatinine concentrations. Still, all meta-analyses concluded that serum cystatin C was superior to urine cystatin C.

## Putting proteinuria analysis into clinical practice

As outlined above, the presence and amount of various proteins in the urine varies considerably across the spectrum of renal disease, in particular in distinguishing patients with an isolated tubulopathy from patients with chronic kidney disease involving the glomeruli and chronic kidney disease of non-glomerular origin (i.e., CAKUT). Here, a limited strategy measuring albumin, α1-microglobulin, and creatinine is able to separate these entities with high sensitivity and specificity [3]. Beara-Lasic et al. confirm that the protein-creatinine ratio does not differentiate between Dent disease and a glomerulopathy, a fact that causes much confusion and has led to unnecessary kidney biopsies [28]. Instead, an α1-microglobulin-creatinine ratio of 120 mg/g had a sensitivity of 86% and specificity of 95% to distinguish Dent disease from other forms of chronic kidney disease, even when analyzing the subgroup of tubulointerstitial disease separately. α1-microglobulin can be substituted by the albumin-total protein ratio (cutoff 0.21 g/g). Still, this reduces specificity, in particular when separating Dent disease from tubulointerstitial disease (specificity 55%). Based on these findings, the low albumin contribution of some 30% of total proteinuria in the case report by Preston et al. [2] published in this issue of *Pediatric Nephrology* argues against FSGS as primary diagnosis in their patient and points towards the diagnosis of Dent disease [28].

Beara-Lasic et al. did not present cutoffs to distinguish chronic kidney disease of tubulointerstitial origin from glomerular disease. These two entities can be separated using urinary albumin and total protein concentrations (AUROC 0.82), whereas the albumin-total protein ratio (AUROC 0.61) and the α1-microglobulin-creatinine ratio (AUROC 0.53) performed poorly. In this setting, α1-microglobulin should be related to total protein (AUROC 0.82) or albumin concentration (AUROC 0.82).

**Fig. 5:**
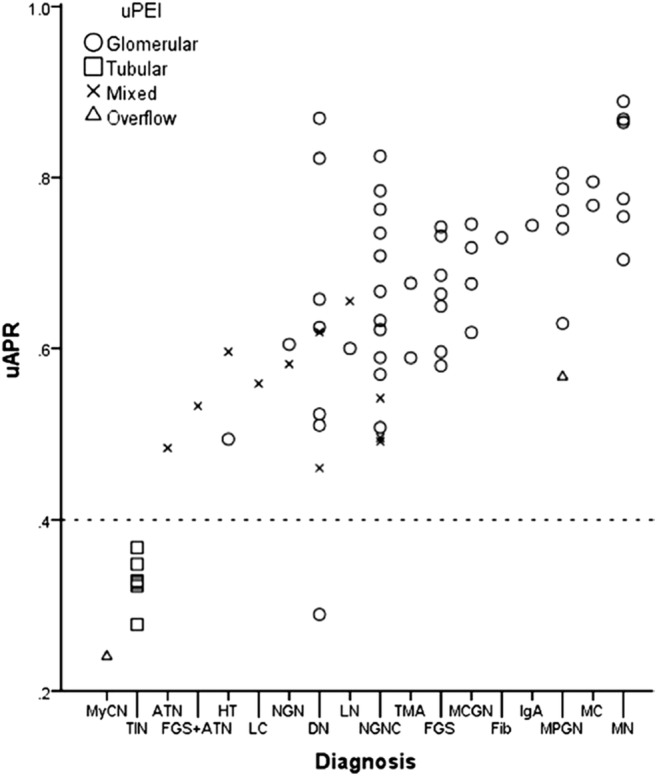
Histological diagnosis and urine albumin/protein ratio. A urine albumin/creatinine ratio (uAPR) value of 0.4 (dotted line) demonstrates a clear distinction between tubulointerstitial disorders and glomerular disorders. uPEI, urine protein electrophoresis and immunofixation; ATN, acute tubular necrosis; DN, diabetic nephropathy; Fib, fibrillary glomerulonephritis; FGS, focal segmental glomerulosclerosis; HT, hypertensive nephrosclerosis; IgA, IgA nephropathy; LC, light chain deposition disease; LN, lupus nephritis; MC, minimal change disease; MCGN, mesangiocapillary glomerulonephritis; MPGN, mesangioproliferative glomerulonephritis; MN, membranous nephropathy; MyCN, myeloma cast nephropathy; NGN, necrotizing glomerulonephritis; NGNC, necrotizing glomerulonephritis with crescents; TIN, tubulointerstitial nephritis; TMA, thrombotic microangiopathy. From Smith et al. [71], reproduced with permission

Smith et al. used the albumin-total protein ratio to distinguish tubular from glomerular proteinuria defined by urine protein electrophoresis and immunofixation in some 1000 urine samples [71]. In their hands, ROC analysis of the albumin-total protein ratio yielded an AUROC of 0.84 and was comparable to the ß2-microglobulin-creatinine ratio. Using a cutoff of 0.40 mg/mg for the albumin-total protein ratio, sensitivity for the diagnosis of tubular proteinuria was 75% and specificity 73%. Figure 5 shows the distribution of the albumin-total protein ratio vs. histological findings. In patients with combined glomerular and tubulointerstitial lesions, albumin-total protein ratio was inversely related to the severity of tubulointerstitial lesions, indicating increasing amounts of LMW proteins as it is unlikely that the shedding of immunoglobulins will account for this change [71]. Most patients with pure glomerular disease had values above 0.60 mg/mg. This is in line with Ohisa’s series of 579 patients (69% with kidney biopsy) where this cutoff had a sensitivity of 97% and specificity of 100% [72].

These findings can also be used in the diagnostics of macroscopic hematuria. Serum albumin constitutes about 55% of total protein in healthy persons [73]. In post-glomerular hematuria, full blood has mixed with urine, therefore the albumin total protein ratio will be about 0.55 mg/mg, whereas a higher value suggests a glomerular origin of hematuria.

## Conclusion

A detailed analysis of proteinuria can provide important diagnostic and prognostic information. These tests are cheap, non-invasive, and rapidly available in most clinical laboratories and an important adjunct to renal biopsy and modern molecular genetic techniques. From a historical perspective, taking a close look at urine was the starting point of laboratory medicine more than 2000 years ago [74].
